# Making decisions in a complex information environment: evidential preference and information we trust

**DOI:** 10.1186/1472-6947-13-S3-S7

**Published:** 2013-12-06

**Authors:** Vetta L Sanders Thompson

**Affiliations:** 1 Brown School, Washington University in St. Louis, One Brookings Drive, St. Louis, MO -63130, USA

## Abstract

**Background:**

Informed decision making requires that those individuals making health and health-care decisions understand the advantages and disadvantages associated with particular health options. Research and theory suggest factors that contribute to the decision-making process: data on the likelihood of risks and benefits, level of certainty about outcomes, familiarity with the health issue, characteristics of information sources and presentation, and patient values and beliefs. As the health information environment increases in complexity, it becomes important to understand how interactions among information sources, family, and friends may affect the processing of health information and choices and their alignment with available evidence.

**Analysis:**

This paper discusses the potential interactions among social networks, information sources and evidential preferences for health information as they influence health decisions. The role of family and friends who increasingly search for health information for others and the potential for information filtering influenced by second- or third-party attitudes and preferences is explored. Evidential preferences suggestive of the potential value of social math (creatively presented statistics) strategies for presenting data, the information-processing factors that may make personal experiences, anecdotes and testimonials that are often shared within social networks and may exert powerful influences on health decisions are examined in this article.

**Conclusions:**

The paper concludes with recommendations for revised health-communication practices, health professional training to improve patient understanding in the clinical encounter, and directions for future research. Simple, direct, and socially relevant communications that avoid conflicts with the values and beliefs of the individual, as well as those of the family and social network, are recommended.

## Background

In a complex health-care system, it is important that patients have the ability to participate in decision making at the level desired. It is equally important that patients be armed with accurate information relative to the issues at hand. For their participation to be feasible and result in outcomes acceptable to them and their families, the information must be accessible and understandable to patients—offering the individual seeking treatment or preventive or palliative care the opportunity to develop a sound understanding of the medical issue(s), the course and progression of the disease or health issue, and the diagnostic and treatment options (including alternatives to standard options) available to them [1].

To make health decisions, research and theory suggest that patients require facts and evidence about disease severity, their susceptibility to the disease or condition, the risks and benefits of screening/tests or treatment, consideration of their values/beliefs, and adequate support for the decision. Patients wrestling with health decisions may receive converging and conflicting information from a range of sources; while it is unclear how information from different sources is weighed and integrated, research and theory suggest that decisions are weighted toward familiar information, personal beliefs and values, and social norms [2,3]. Lack of knowledge or uncertainty about any of the relevant issues may result in failure to explore options and engage fully in health decision making, increasing the likelihood of deference to clinical professionals and the health system, other experts in their networks, and friends and family—ultimately heightening the likelihood of patient dissatisfaction with care choices made [4].

As the United States attempts to confront the challenges of rising health-care utilization and costs, imposed in part by an aging population and in part by failures to implement system efficiencies, those engaged in health decision making are confronted with an ever-changing information environment. Patients must integrate information from health-care professionals and significant others with information obtained via technology and through the news and entertainment media. In such an information-rich environment, it is not uncommon for only a few general characteristics of data to be evaluated by patients and/or caregivers. Often such characteristics include the extent to which the data fit with existing beliefs, were obtained from credible sources, and are believed by important others [3].

While cognitive and emotional processing of information related to health decisions is an individual activity, social norms and factors in the social environment are very likely to influence information availability and access, the patient’s sense of susceptibility to a disease or condition, and confidence in their abilities to make a good decision. In this context it may be worthwhile to highlight the importance of descriptive social norms that educate via shared social information [5]. Because of the important role of family, friends, and the media in demonstrating social norms, it is important to understand the involvement of these entities in how patients acquire and process health information.

This paper discusses the role of information sources and evidential preferences— including the influence of health professionals, general and ethnicity-specific media, Internet sources, personal experience, and family and friends and their testimonials and anecdotal evidence—in health decisions. Recent research reviewing the role of media and evidential preferences in ethnic communities, particularly studies examining social attitudes and influences, is explored to facilitate understanding of preventive health decisions. The paper concludes with recommendations for: (a) revised health communication practices, (b) health professional training to improve patient understanding and participation in the clinical encounter, and (c) future research directions focused on the issues discussed.

## Analysis

### Sources of health information

Although health professionals, particularly physicians, are influential [6,7] and trusted sources of health information [8,9], family and friends also provide health information and affect health decision making. Family and friends influence beliefs and attitudes about health and the use of health-care resources, particularly among patients who are less educated and nonwhite [10]. Family and friends may influence the clinical encounter, interpreting health information obtained from a variety of sources. The extent to which this is the case is suggested by data from the 2000–2004 Cancer Information Service call service; approximately one-third of callers indicated that they were the spouse, relative, or friend of a patient with diagnosed cancer [11].

Recent surveys indicate that 80% of Internet users, or 59% of the U.S. adult population, has searched for health information online [12]. Despite this fact, we know that some groups are more likely than others to use the Internet for health information. The 2009 National Health Interview Survey indicated that among adults 18 to 64 years of age, non-Hispanic white people, those with higher incomes, and those who were employed were more likely to have used the Internet for health information than were individuals from groups with other demographic characteristics [9].

Media are important sources of health information, and professionals involved in patient care must recognize that media sources accessed by different patients and health product consumers reflect diversity in the same way as the populations that access these sources [13-17]. Members of underserved communities trust and value ethnicity-specific media and the health-related coverage provided through ethnic media outlets must be included in analyses of the health information environment [14,15,17]. This issue is made more salient by reported variations in the ways in which individuals from different racial and ethnic groups access news and information important to their lives.

Asian Americans reportedly express a preference for print materials on health issues [15]; use of media for health information among Hispanic populations has been shown to vary by acculturation [16]. African American newspapers have been shown to be effective in broadening the reach of cancer messages, and readers trust those newspapers for health and cancer information [17]. With so many information sources and the documented variations among individuals and communities in terms of media preferences, knowledge about the sources people consult and how they respond to health information has become a topic of increasing interest to people involved in efforts to promote improved health through enhanced patient engagement and active participation in health care.

### Health information use and processing and the presentation of evidence

Rosenstock notes that a “person’s beliefs about the availability and effectiveness of various courses of action, and not the objective facts about the effectiveness of action, determines what course he/she will take” ([10] p.7). These beliefs are affected by the descriptive and injunctive norms (perceived approval/disapproval) of the individual’s social/referent group [10], as represented by family, friends, and media sources. Health professionals are relied upon to communicate with patients and their families about the evidence base that guides clinical health practice. However, practitioners may have limited knowledge of the information sources accessed, the content of the information, and effective communication strategies with patients and patient groups who most need this information [10,18].

Differential patient and family response to health information may be related to demographics, cognitive and emotional characteristics, and the differences in the information environments that patient, family, and physician encounter. Diversity in health literacy [19] also affects how health information might be effectively presented [13,18]. Too often there is a mismatch between the individual patient and family members’ skills, needs, preferences, and expectations and the information and services that are available [18]. Data suggest that this mismatch is most often encountered by individuals who have a low income, are less well educated, are older, and are members of racial and ethnic groups [18,19]. Current evidence suggests the need to explore how this mismatch contributes to discrimination, implicit bias, and disparities in professional communication of health information and services [20].

Johnson’s Comprehensive Model of Information Seeking suggests that the demographic characteristics of patients and their family and friends, personal experiences, beliefs and the salience of the topic will affect perceptions of information sources and the utility of the information [21]. Dervin noted how situational circumstances affect the strategies used to decide when, what, and how to use information, including health information [22]. These issues can be complex, and at various times there may be the need or desire for facts or information from authorities; from family members, peers, or supportive others; or some combination of these sources. Among health information seekers, situational circumstances might refer to lived experiences, such as previous interactions with health-care professionals and systems, family history, and previous experiences with disease. For example, individuals whose family members have died of cancer may view cancer survival statistics differently than those with no family history of cancer deaths. In addition, patients may have preferences for particular styles and content when evidence is presented. For example, individuals vary in their preference for graphical versus numerical statistics and narrative formats.

Whether it is the patient, a family member, or friend, people facing important health decisions may feel overwhelmed or numb in the face of statistical data, which can interfere with information processing and participation in health decisions [4]. For this reason, it is important to understand how the presentation of evidence influences peoples’ responses to health information [23]. *Evidential* approaches to health communication present data and statistics on disease prevalence, the need for physical activity, healthy eating and screenings, the effectiveness of screenings, diagnostic tests and treatment options, and/or effects of the disease, screening strategy, or treatment to a given group or population [23,24]. The presentation of evidential information is an attempt to raise awareness, concern, and/or perceived personal vulnerability to a health issue or concern. The appropriateness of the evidential presentation is believed to affect acceptance and willingness to act on information. Evidence can be statistical or nonstatistical, (e. g., cases presented by professionals, testimonials or anecdotes from peers). Statistical evidence can be presented using general statistics such as percentages or rates and standard metrics such as mortality and morbidity. Data can be transmitted with visuals or using strategies such as social math/creative epidemiology [25]. Social math presentations describe statistics using examples drawn from the environment and are likely to be familiar and relevant to the user. An example is: “Imagine yourself and two family members. One of you is likely to be diagnosed with some form of cancer during your lifetime.” In addition, it is important to consider how the evidence is framed. Evidence may be presented by highlighting positive outcomes (gains from choosing screening and early detection) or negative outcomes (losses from choosing not to screen or engage in early detection strategies) for the individual or the referent social group [25,26]. Nonstatistical evidence may take the form of anecdotes, testimonials, or stories.

Lipkus et al. confirmed the importance of presenting risk information to those making decisions about health behaviors [27]. This research suggests that risk information can be presented to increase perceived risk without increasing worry, fear, or anxiety. In attempting to understand how risk information should be presented, Royak-Schaler found a preference for information that addressed family history and personal risk [26], which may be related to the values that lead to the importance of family in health decisions. Arkes and Gaissmaier noted a preferential response to information presented in graphic versus quantitative forms [28]. However, some data suggest the need to consider how factors, such as perceived ambiguity of cancer screening guidelines, might affect health attitudes (worry, fear, and anxiety) after risk information is presented [29].

One component in the understanding of how evidence presentation affects the reach and relevance of health communications is knowledge of community reactions to the presentation of health statistics [23]. Thompson Sanders et al. reported on efforts to gain insight into these reactions using data from nine focus groups with African American adults (three female groups [N = 17] and six male groups [N = 32]) presented with cancer-related evidential statements [23]—statements provided general statistical data, ethnicity-specific statistics, statistics highlighting disparities, and social math examples (creative epidemiology) and statements that provided positive and negative framing (survival and mortality data) [24].

Comments made by the focus group participants suggested the use and influence of health information participants obtained themselves, as well as the influence of their families in decisions about health [23].

“I know I have a lot of things that are wrong with me because I do a lot of research.”

“You need someone in your family who loves and supports you so … you know, we’ve been going to this physician for so and so time. So, now what kind of diagnosis, what’s the prognosis? And then what’s the next step? And if nothing has really been done positive, the other person might say, why don’t we go get a second opinion?”

When presented with the typical statistical presentation on a disease and its likely course, the focus group participants related their experiences and behaviors to the presented information and expressed a desire for more information related to the disease, including steps for health behavior change [23]. Participants dismissed data presented as estimates or approximations of rates of disease. Consistent with past research [24,30], ethnicity-specific statistics, designed to increase identification with targeted materials, helped participants to see themselves as a part of a high-risk group, emphasizing the importance of the referent group. Disparity data, focus on how health behaviors and outcomes differ among demographic groups (race/ethnicity, income, sex, region or urban/rural status), are relevant to the discussion of how to present health information. Among stigmatized populations, how such data are presented can affect attitudes and raise suspicions about recommendations and treatment before any contact is made with health-care professionals. When presented with disparity data, the focus group participants expressed negative emotions and feelings of mistrust and engaged in discussions of the motives for compiling the data presented, again questioning source credibility. A similar but less intense response was noted in discussions of ethnicity-specific health statistics. In contrast, statistical evidence that was presented with social math strategies was seen as more personal, with participants expressing a preference for statements referencing family [23]—once again suggesting that consideration be given to the contributions of family members in health information processing. In a study by Nicholson et al., the presentation of racially comparative cancer information indicated that participants exposed to disparity articles reported less intention to be screened for colorectal cancer [25]. The effects were more intense for individuals identified as highly mistrustful and provide further evidence of the importance of considering community responses to the presentation of evidence.

### Anecdotes and personal experiences as evidence

Testimonials and statements related to personal, family, or group experience can also be used as a form of evidence and may readily be shared among family members and friends. Testimonials are usually compelling and easy to understand on emotional, as well as a cognitive, levels and can influence responses to health behavior guidelines and treatment choice [23,24]. Studies have shown that anecdotes are also a powerful form of evidence and can influence a person’s belief about how a health behavior, disease, or treatment affects him or her through the experience of similar others (descriptive norms) [5]. Importantly, this form of evidence is readily shared within social networks that include family. The emotional arousal that is associated with this form of information contributes to the willingness of members within the social network to pass this information along [3].

The available evidence suggests the potentially strong influences of personal experience, anecdotes, or testimonials shared among family members in the processes that contribute to health-related decision making. Fagerlin et al. illustrated the power of anecdote on treatment choice [31]. The researchers provided identical statistical data on treatment effectiveness to two groups but varied the representativeness of the anecdotes accompanying the data presentations. Based on the treatment selections made, the anecdotes had a significant impact on treatment choice, even when identical statistical data were presented. The strong influence of anecdotes on health decision making may be because their use allows individuals to identify the recipients of treatment and provide a sense that those experiencing the disease are known [28]. Slovic suggests that the sense of familiarity created by anecdotes enhances the sense of concern for and about the health issue [32]. In addition, this sense of familiarity may allow the information to be processed more easily [3]. While anecdotes and testimonials are powerful in their own right, additional data suggest the importance of considering the effects of combining these forms of evidence with statistical data [33].

### Processing health information

Lewandowsky et al. noted that individuals tend to accept the truth of information, unless there is significant distrust, the message is implausible, and significant attention is applied in the situation in which the information is received [3]. The importance of the information to the individual will also affect the extent to which new information is evaluated sufficiently. Even when attending to and attempting to evaluate new information, there is a tendency to draw on prior knowledge and to consider how the new information fits with that knowledge and the individual’s beliefs. Individuals express more doubt about information that is inconsistent with their experiences. When familiar and consistent with experience and beliefs, erroneous information may be accepted and used in decision making—information that may be difficult to displace, even in the presence of deliberate efforts to correct misinformation and provide correct and relevant information in its place [3].

Furthermore, while individuals reflect on their experience in terms of harm or benefit, information that would allow appropriate comparisons between experience and evidence is typically not readily available. It is difficult to avoid bias when the decision maker lacks access to some component of the data, whether harmful or beneficial. Patients attempting to decide on adherence to health guidelines and treatment recommendations often do not have information on potential harms associated with a treatment or product; typically, more detailed information is available to describe possible benefits [28]. Personal and family experiences are particularly problematic in this respect, with only one aspect of experience available through stories and anecdotes. Beliefs and attitudes related to health issues are necessarily communicated from this single perspective. This is often the case when harmed family members, close friends, and influential others are unable to articulate how they might have benefitted from a treatment or screening. Moreover, if benefitted, these individuals may be unable to describe the side effects of a screening or treatment. In either case, family and friends are confident in their affective as well as cognitive presentations of their experiences and opinions [28]. Because the stories and anecdotes shared within a social network suggest important social norms and attitudes and are delivered by trusted sources, they are powerful and difficult to overcome as important components of decisional processes that affect many people’s choices about the health services they receive.

## Conclusions

Social psychology and health communication research provide insights into how and why the information environment is becoming more challenging for those attempting to promote adherence to treatment recommendations and guidelines and to engage patients more fully in their own care. Patients and their family members and social networks have greater access to health information from a variety of sources and can share their experiences and preferences rapidly using ever expanding media and technologies [8,9,11,12]. Members of diverse communities have different preferences for information media and how evidence is presented [13-17,23,24], with some presentation strategies provoking strong and important emotional responses. It is important to continue research on these issues and changes in the health communication environment.

Consistent with current research and recommendations, key components in effective health professional communications with patients will (a) deliver simple, direct, and relevant health information, b) affirmatively state facts, while providing alternatives to information that may be inaccurate, (c) consider strategies to engage family and friends consistent with patient preference, (d) use graphic and other creative presentations of statistics, as well as anecdotes and testimonials, and (e) consider relevant social norms, evidential preferences, and sources within diverse populations [3,23,28,30,31]**.** While physicians are trusted health information sources, personal and family experiences and beliefs—as well as anecdotes and testimonials encountered in the media or through social networks—exert powerful influences in decision processes [3,31]. It is important that health professionals be trained and prepared to listen to patients and discuss their experiences and their perspectives on evidence—however it was acquired—as these affect their health decisions. Conversations addressing screening and treatment options must balance evidential data with consideration of patient and family attitudes, values, beliefs, and descriptive social norms, which may at times conflict with each other, in addition to treatment recommendations [23,30]. Furthermore, health and health-care guidelines are based on population data, and the meaning of such data for any individual is dependent on how closely his/her health and risk profile matches that of the general population. This fact is not well understood and is not usually a part of the discussion as health professionals share evidence and recommendations with individual patients and their family members [28]. The implications of applying data at different levels must be presented in ways that do not unintentionally undermine confidence in the data available to the patient or significant others [23]. While this review has focused heavily on cognitive processing of information, stronger integration of the influence of emotions in processing evidence will strengthen efforts to communicate health information.

Given what we know about evidential preferences [23-25], increased media use of social math and graphically displayed risks/harms and benefits of health treatments and behaviors are strategic. What remains to be explored is how these presentation strategies can be integrated into clinical conversations that promote understanding by all parties—assisting the patient in making appropriate evidence-based decisions and facilitating the clinician’s understanding of patient values and preferences and their importance in choosing a course of action. Despite good intentions, clinician attempts to foster healthy skepticism and promote exploration of health beliefs informed by information from particular sources might, in fact, result in increased suspicion of the proposed sources of information. Similarly, over-reliance on reported health disparity data in an attempt to motivate patient engagement and/or other action may increase suspicion among patients concerning treatment approaches for which fairly strong evidential data are available [23,25]. Technology-based health information platforms integrated into physician offices offer an expanding array of possibilities, but research is needed to generate data that will help in striking the correct balance of consumer-targeted health information and health professional/patient discussion in the health-care setting.

While family and friends have an important role to play in health decisions, we know surprisingly very little about which diseases, treatments, and life circumstances affect when and how patients are willing to include family members in decision-making processes. Future research should examine these issues, factors affecting the willingness of family and friends to be involved, and the sources/types of information they might find useful to health decision making. Consistent with comprehensive models of seeking and using health information [21], future research should examine the characteristics of traditional (person to person, print, radio, television, etc.) versus technology-based media (kiosks, the Internet, including social media) that may influence the active and passive acquisition and use of health information. It is important to understand how to leverage Internet and population-specific media to increase the likelihood that individuals encounter statistical and narrative presentations that do not violate personal and social norms and, thereby, make evidence presented in support of recommendations and guidelines that may be changing (often fairly dramatically, as was the case with the recent update of breast cancer screening guidelines) easier to assimilate. While the issue of how health professional-patient conversations are best conducted remains an area ripe for further research, research should also consider how we best facilitate conversations related to health decisions within the family and influential other networks. For example, how can we facilitate timely discussions of health decisions between patients and their family members? How do we address differences in perspectives related to variations in cognitive and emotional processing that will likely emerge as patients and family members face health decisions together?

Studies that identify which family, friends, and members of the social network are likely to need health information to assist in decision making about preventive health, treatment, and palliative care issues and under what conditions will be important to improve adherence to recommended standards of health care. To assure equity of access, future research should evaluate access, acceptance, and utility of sources of information in various contexts, populations, and health concerns. Finding the right balance and developing the right tools to promote optimal patient engagement and foster understanding of evidence in ways that do not discount or undermine important individual and culture-based values and preferences is still a work in progress. Additional research will advance the agenda in moving us toward the proper balance.

## Competing interests

The author declares that she has no competing interests.

## Declarations and disclaimer

The Eisenberg Conference Series 2012, *Supporting Informed Decision Making When Clinical Evidence and Conventional Wisdom Collide*, was conducted in Rockville, Maryland, by the John M. Eisenberg Center for Clinical Decisions and Communications Science, Baylor College of Medicine, Houston, Texas, for the Agency for Healthcare Research and Quality under Contract No. HHSA 290-2008-10015-C. Publication costs for this supplement were funded by this contract. The author of this article is responsible for its content. No statement may be construed as the official position of the Agency for Healthcare Research and Quality and of the U.S. Department of Health and Human Services.

This article has been published as part of *BMC Medical Informatics and Decision Making* Volume 13 Supplement 3, 2013: Articles from the Eisenberg Center Conference Series 2012: Supporting informed decision making when clinical evidence and conventional wisdom collide. The full contents of the supplement are available online at http://www.biomedcentral.com/bmcmedinformdecismak/supplements/13/S3.
